# Assessment of the Microbial Constituents of the Home Environment of Individuals with Cystic Fibrosis (CF) and Their Association with Lower Airways Infections

**DOI:** 10.1371/journal.pone.0148534

**Published:** 2016-02-09

**Authors:** Alya Heirali, Suzanne McKeon, Swathi Purighalla, Douglas G. Storey, Laura Rossi, Geoffrey Costilhes, Steven J. Drews, Harvey R. Rabin, Michael G. Surette, Michael D. Parkins

**Affiliations:** 1 Department of Microbiology Immunology and Infectious Diseases, University of Calgary, Calgary, AB, Canada; 2 Department of Biological Sciences, University of Calgary, Calgary, AB, Canada; 3 Department of Medicine, The University of Calgary, Calgary, AB, Canada; 4 The Department of Biochemistry & Biomedical Sciences, McMaster University, Hamilton, ON, Canada; 5 Department of Laboratory Medicine & Pathology, University of Alberta, Edmonton, AB, Canada; Lee Kong Chian School of Medicine, SINGAPORE

## Abstract

**Introduction:**

Cystic fibrosis (CF) airways are colonized by a polymicrobial community of organisms, termed the CF microbiota. We sought to define the microbial constituents of the home environment of individuals with CF and determine if it may serve as a latent reservoir for infection.

**Methods:**

Six patients with newly identified CF pathogens were included. An investigator collected repeat sputum and multiple environmental samples from their homes. Bacteria were cultured under both aerobic and anaerobic conditions. Morphologically distinct colonies were selected, purified and identified to the genus and species level through 16S rRNA gene sequencing. When concordant organisms were identified in sputum and environment, pulsed-field gel electrophoresis (PFGE) was performed to determine relatedness. Culture-independent bacterial profiling of each sample was carried out by Illumina sequencing of the V3 region of the 16s RNA gene.

**Results:**

New respiratory pathogens prompting investigation included: *Mycobacterium abscessus*(2), *Stenotrophomonas maltophilia*(3), *Pseudomonas aeruginosa*(3), *Pseudomonas fluorescens*(1), *Nocardia* spp.(1), and *Achromobacter xylosoxidans*(1). A median 25 organisms/patient were cultured from sputum. A median 125 organisms/home were cultured from environmental sites. Several organisms commonly found in the CF lung microbiome were identified within the home environments of these patients. Concordant species included members of the following genera: *Brevibacterium*(1), *Microbacterium*(1), *Staphylococcus*(3), *Stenotrophomonas*(2), *Streptococcus*(2), *Sphingomonas*(1), and *Pseudomonas*(4). PFGE confirmed related strains (one episode each of *Sphinogomonas* and *P*. *aeruginosa*) from the environment and airways were identified in two patients. Culture-independent assessment confirmed that many organisms were not identified using culture-dependent techniques.

**Conclusions:**

Members of the CF microbiota can be found as constituents of the home environment in individuals with CF. While the majority of isolates from the home environment were not genetically related to those isolated from the lower airways of individuals with CF suggesting alternate sources of infection were more common, a few genetically related isolates were indeed identified. As such, the home environment may rarely serve as either the source of infection or a persistent reservoir for re-infection after clearance.

## Introduction

CF has traditionally been viewed as a disease of limited pathogens. Classical pathogens such as *Staphylococcus aureus*, *Pseudomonas aeruginosa*, *Burkholderia cepacia* complex *(Bcc)* and *Haemophilus influenzae*, have been well characterized [1]. Increasingly these are being supplemented with “emerging organisms” such as *Stenotrophomonas maltophilia*, *Achromobacter* spp, and nontuberculous mycobacteria [2,3]. Many of these organisms have been found to develop chronic infection and result in an accelerated clinical decline [2,4]. However, due to the increasing use of high throughput culture-independent molecular techniques, scientist have identified a diverse number of “newly appreciated” organisms infecting the CF lower airways [5–8]. Many of these organisms are thought to effect clinical outcomes either through direct or indirect pathogenic potential [9–11]. The diversity of the organisms colonizing and infecting the lower airways is termed the CF lung microbiota [5,7,10,12,13].

Where the constituents of the lung microbiome are derived is relatively unknown. For certain pathogens such as *P*. *aeruginosa* and *Achromobacter xylosoxidans* they are ubiquitously distributed in natural environments (water, soil, decaying matter) and their infrequent role as human pathogens (being opportunistic), led to the presumption that most patients acquired these infections from environmental reservoirs [2,14]. Indeed, in CF patients with newly identified lower airways infection with *P*. *aeruginosa*, a home environmental reservoir with a genotypically identical strain could be identified in 18% of patients, most frequently identified in the bathroom [15]. In addition, shared strains of organisms such as *P*. *aeruginosa* and *S*. *aureus* in unrelated patients have led researchers to identify that limited transmission amongst patients is possible if adequate infection controls are not in place [16].

A critical innovation within CF care has been the recognition that chronic infection can be terminated thereby potentially avoiding the resultant clinical sequelae (termed “eradication”) [17,18]. Indeed, with the early recognition and treatment of new infections many new pathogens can be eradicated. Unfortunately, success is not always possible and some patients will fail antibacterial therapy, and others will be re-infected [19]. This is possibly due to repeated exposures from a persistent latent reservoir that has not been eliminated. The goals of our research were to define the microbiota of the home environment of individuals with CF and to determine if the microbiota of their home environment shares microbial constituents with the lower airways. In CF patients who acquire “new” infection, it is possible that the source of these “new” infections exists within their homes. If so, these sources may potentially serve as a persistent latent reservoir for re-infection, even after successful eradication therapies.

## Methods

### Patients and Sample Collection

CF patients with new bacterial infections identified from sputum analysis collected during routine quarterly appointments at the Southern Alberta Adult CF clinic were approached for inclusion. If the patient consented, an investigator organized an appointment to sample the patient’s home within two weeks of the original sputum sample being collected. During the visit, the investigator would, using aseptic techniques, sample multiple sites in the patients homes including; showerheads, bathroom/kitchen sink faucets and drains, humidifiers, clothes washers, as well as CF specific equipment including airway clearance devices, compressors, and nebulizers, as CF pathogens have been previously found to exist in such niches and where applicable recreational drug equipment (not medically sanctioned) was similarly sampled [15,20–22]. An average of 6–8 sites were sampled within the homes of each patient using a COPAN eSwab (Thermo Scientific) anaerobic transport media. A repeat sputum sample was collected simultaneously in sterile containers and transported to the research laboratory within one hour using GasPak anaerobic transport systems (BD Diagnostics) (<0.01%,O_2,_ 10% CO_2_). Ethics for this study was approved by the University of Calgary Conjoint Health Research Ethics Board (CHREB 14–2462) and each patient provided written informed consent.

### Definitions

To be included patients had to meet the established definition of CF [23]. Stage of lung disease was defined based on spirometery as measured by the forced expiratory volume in one second (FEV_1_); advanced <40%, moderate 40–70%, mild 70–90%, and very mild >90% predicted. Chronically colonizing pathogens were defined using previously described definitions [24]. If organisms were present in ≥50% of a minimum of four sputum cultures collected from patient in the preceding year they were consider chronically infected. Organisms deemed newly infecting, thereby prompting investigation, were defined in individuals who had not previously grown the same organism.

### Culture-Dependent Studies

Within one hour of collection, traditional microbiological protocols were used to cultivate organisms. Sputum was physically sheared, vortexed and 100 μL was serially diluted from 10^−1^ to 10^−5^ in Brain Heart Infusion (BHI) broth (Difco) in an anaerobic chamber (5% CO_2_, 5% H_2_, balanced N_2_). Similarly, environmental swabs collected in Amies transport media were serial diluted in BHI broth. Aliquots of 100 μL of the 10^−2^ and 10^−4^ serial dilutions of the environmental, and 10^−3^ and 10^−5^ dilutions of the sputum were plated on differentially selective media types. Samples were plated on a range of general and semi-selective medias including; Fastidious Anaerobe Agar (FAA), Chocolate (CHOC) (Difco), Columbia Blood Agar (CBA) (Difco) with 5% defibrinated sheep blood (Materials Bio Labs), and incubated for seven days anaerobically at 37°C. Samples were similarly plated on Trypticase Soy agar (TSY) (Difco), Brain Heart Infusion (BHI) and Colistin supplemented BHI (BHI Co) (Difco), Columbia Naladixic Acid (CNA) (Difco), Mannitol Salt Agar (MSA) (Difico), Pseudomonas Isolation Agar (PIA) (Difco), McKay Agar,[9] and MacConkey (MAC) (Difico) and grown aerobically with 5% CO_2_ at 37°C and incubated for 48 hrs. For the two patients where *M*. *abscessus* was identified, an additional culturing step on Middlebrook 7H11j solid medium at 30°C for 12 weeks was performed [25]. Unique isolates were selected for based on differences in colony morphologies and purified in triplicate, assigned a study ID and stored in 10% Skim milk (Difico) at -80°C for later assessment.

### Bacterial identification

Cells from uniquely identified isolates were grown under appropriate conditions for at least 24 hours, and then re-suspended in 200 μL of sterile water and boiled for 20 min. PCR amplification of the 16S rRNA gene was carried out using the boil preparation from the target isolate (2 μL of the supernatant was used as template for PCR amplification of the 16S rRNA gene) [26]. The oligonucleotide primers used, 8F and 926R, were previously described by Sibley et al., [6]. Samples were sent for Sanger sequencing to Macrogen (Seoul, Korea). Taxonomic assignment of cultured organisms was achieved using a combination of databases including, NCBI BLAST, Ribosomal Database Project (RDP) classifier, and Human Oral Microbiome Database (HOMD) [27]. If match ID was ≥ 97% organisms were identified to the species level [27–29]. Organisms with sequence identity >95% but <97% to known or well characterized 16S rRNA sequences were resolved to the genus level, 90–95% to the family level, 85–90% to the order level, 80–85% to the class level, and 77–80% to the level of phyla [28–30].

### Bacterial Strain typing

Where concordant positive cultures for organisms within the home environment and sputum were identified, bacterial strain typing was performed using pulse-field gel electrophoresis (PFGE). Strains were incubated from frozen stocks onto TSY agar and cultured at 37°C for 24 hours. PFGE protocols were performed as per Parkins et al., and Workentine et al. [31,32],. Cell suspensions were made in a cell suspension buffer (CSB) (1M NaCl, 10mM Tris-HCl, pH7.6). Next PFGE plugs were made by adding equal volume of 1% (w/v) SeaKem Gold Agarose in TE_1_ buffer (10mM Tris-HCl, 1mM EDTA, pH 7.6) to 150uL of CSB in a plug mold (BioRad). Plugs were left to solidify in cast at room temperature (RT) for 15 minutes and then incubate at 37°C for 4 hours in a tube containing 1mL of cell lysis buffer (CLB) (1M NaCl, 100mM EDTA[pH 7.5],0.5% Brij-58, 0.5% Sarcosyl, 0.2%Deoxycholate, 6mM Tris-HCL[pH 7.6], l mg/mL lysozyme powder, 20ug/mL RNase A). Next plugs were transferred to a tube containing 1 mL ESP solution (0.5M EDTA [pH 9], 1% sarcosyl, 70 μg/mL proteinase K) and incubated in a water bath at 50°C overnight. Plugs were then washed 6 times using TE_0.1_ buffer (T10 mM Tris-HCl, 0.1mM EDTA, pH 7.6) and stored in the TE_0.1_ buffer at 4°C. This was followed by restriction digestion of the isolates. Plugs were rinsed with 1X reaction buffer (NEBuffer Cutsmart) for 15 min at RT, then digested with specific restriction enzymes (New England Biolabs) for 4 hours at 37°C. Different restriction enzymes were used for the various organisms being studied based on a review of previously published work with few modifications [31,33–38].

Assessment of strain relatedness was performed using the CHEF Mapper system (BioRad). Lambda ladder PFG Marker (New England Biolabs) was used as a standard. Strains that had banding patterns that were ≥80% identical were considered related, conforming to the Tenover criteria, where isolates with up to 3 band differences are still considered related. PFGE profiles were compared using BioNumerics, version 7.0 (Applied Maths, Austin, TX) [39]. PFGE banding profiles were compared using the Dice coefficient, allowing for 2% tolerance in band matching.

### Culture-Independent Studies

Microbial community profiling of environmental and sputum samples was conducted as previously described [40,41] In brief, 300 μL of sputum sample was suspended in 800 μL of 200 mM NaPO_4_, 100 μL of guanidine thiocyanate–ethylenediaminetetraacetic acid–Sarkosyl. Similarly for samples collected from environmental reservoirs a COPAN eSwab containing 1 mL of liquid amies solution, was used to collect these samples. These samples were rigorously vortexed and 300 μL of this solution was suspended in 800 μL of 200 mM NaPO_4_, 100 μL of guanidine thiocyanate–ethylenediaminetetraacetic acid–Sarkosyl. The solutions were then homogenized using 0.1-mm zirconia/silica beads (BioSpec Products Inc.).Enzymatic lysis was performed in two steps. Firstly, 50 μL lysozyme (100 mg/mL), 50 μL mutanolysin (10 U/ μL), 10 μL RNase A (10 mg/ml), was added to each sample followed by an incubation at 37°C for 1.5 hours. In the second lysis step 25 μL 25% sodium dodecyl sulfate, 25 μL proteinase K, and 62.5 μL 5 M NaCl was added to each sample followed by an incubation at 65°C for 1.5 hours. Samples were then pelleted via centrifugation at 12 000 × rpm. Next 900 μL of the supernatant was transferred to a tube containing 900 μL phenol-chloroform-isoamyl alcohol (25:24:1) centrifuged at 13 000 rpm for 10 min. The aqueous solution was transferred to a tube containing 200 μL of DNA binding buffer (Zymo). The solution was then transferred to a DNA column (Zymo), washed, and DNA eluted using ultra pure H_2_O.

Next, amplification of the V3 hypervariable region of the 16S rRNA gene was carried out using reverse and forward barcoded primers using the Illumina MiSeq technology at the McMaster Genome Facility (Hamilton, ON) [41]. Sequencing reads were analyzed using custom Perl scripts [41]. Sequences with low quality reads were trimmed using Cutadapt [42]. Next, PANDAseq was used to assemble paired-end reads [43]. Operational taxonomic units (OTUs) were picked using AbundantOTU1 for OTUs with >97% identity [44]. The RDP classifier was used to assign taxonomy against the Greengenes reference database [27,45]. Next, analysis was carried out using QIIME and Phyloseq R package [46,47]. A total of five samples with <1500 reads/sample were considered low quality and were excluded from the study.

## Results

**Fig 1:**
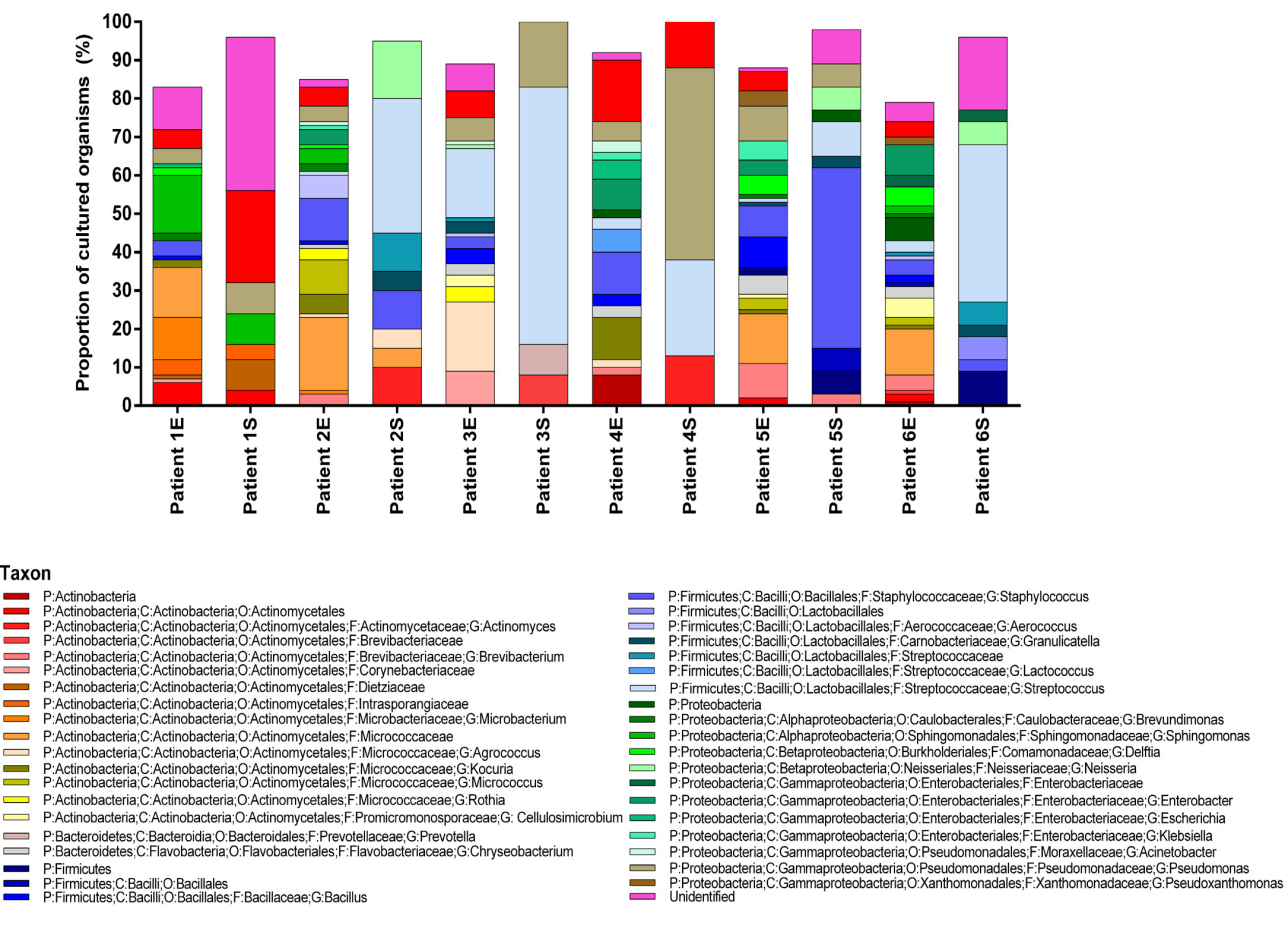
Cultured bacteria recovered from home environment represented as (Patient # 1–6) and source of isolate (S = sputum, E = environment) for all 6 patients. Legend shows a color representation of the taxonomic identification at the various taxonomic ranks from Sanger sequencing of the 16S rRNA DNA of the organisms. Organisms that were cultured in ≥ 6% of the total cultured organisms across all patients are included.

As per the *a priori* defined study cap, a total of six patients with CF and new lower airways infection were enrolled. Patient demographics are detailed in Table 1. Sputum and environmental samples were collected from moist sites in patient’s homes including kitchen/bathroom sink faucets and drains, showerheads, outside faucets, respiratory care equipment, humidifiers and any relevant recreational drug equipment (Table 2). The total number of cultured, morphologically distinct individual bacterial isolates collected from the home environment of each of the six patients using Sanger sequencing ranged from 64–168 (Table 2). By far, sink faucets and drains were the sites with the greatest burden of different microorganisms found in the home environment for all six patients ranging from (53–85%) of the morphologically distinct organisms found in each home (Table 2). Humidifiers were less likely to be contaminated with cultured bacterial organisms compared to other environmental sites, with only (4–5%) of organisms collected coming from that particular sample type. The percentage of organisms found in showerheads varied from patient-to-patient ranging from 1–30% of environmental organisms (Table 2). Between 8 and 32 macroscopically differentiable organisms were identified in each of the six sputum samples (Table 2). Multiple concordant organisms were found to exist in both home and sputum samples (Fig 1 and Table 3).

**Table 1:** Characteristics of patients who underwent correlation of home and sputum microbiome studies.

Patient ID	P1	P2	P3	P4	P5	P6
**Gender**	M	M	M	F	F	F
**Age (years)**	21	33	38	23	77	44
**Genotype**	F508del / F508del	F508del/ G551D	F508 del/ F508del	F508 del/ F508 del	F508 del/ 3849+10kbC->T	F508 del/ F508 del
**Lung Disease Stage**	Very mild	Mild	Advanced	Mild	Moderate	Advanced
**Normal**	MS*SA*	MS*SA*	*PA*	MS*SA*	*SA*	MS*SA*
**Chronic**	*HI*				*PA*	
**Flora**						
**Organism Prompting Investigation**	*PA*, *NC*, *MA*	*PA*, *SM*	*MA*	*PA*, *SM*	*PF*, *SM*	*AX*

MSSA = Methicillin sensitive Staphylococcus aureus, HI = Haemophilus influenzae, PA = Pseudomonas aeruginosa, NC = Nocardia cyriacigeorgica, MA = Mycobacterium abscessus, SM = Stenotrophomonas maltophilia, AX = Achromobacter xylosoxidans, PF = Pseudomonas fluorescens, M = male, F = Female

**Table 2:** Number of morphologically distinct bacterial organisms in the home environment as a function of reservoir.

	Patient ID					
	P1	P2	P3	P4	P5	P6
	(%)	(%)	(%)	(%)	(%)	(%)
**Total Sputum**	25	20	24	8	32	32
**Natural reservoir**						
Shower heads	52/170 (31)	18/139 (13)	4/71 (6)	14/64 (22)	7/110(6)	8/147 (5)
Sink faucets and drains *	97/170 (57)	75/139 (53)	51/71 (72)	49/64 (77)	94/110 (85)	120/147 (82)
Airway clearance device	N/A	10/139 (7)	N/A	N/A	N/A	9/147 (6)
Nebulizers	N/A	22/139 (16)	N/A	N/A	9/110 (8)	2/147 (1)
Compressors	N/A	15/139 (11)	N/A	1/64 (1)	N/A	N/A
Humidifier	7/170 (4)	N/A	3/71 (4)	N/A	N/A	8/147 (5)
Recreational drug equipment ^	14/170 (8)	N/A	N/A	N/A	N/A	N/A
Washer	N/A	N/A	13/71 (18)	N/A	N/A	N/A
**Total Environmental**	170	139	71	64	110	147

(*) Represents various sites of sink faucets and drains throughout the homes of patients. N/A indicates site was not sampled for particular patient. (n) = total number of bacteria organisms cultured from home environment.

(^) Recreational drug equipment represented a hashish pipe.

**Table 3:** The number of concordant isolates found in both sputum (S) and home environment (E) for patients 1–6, using culture-dependent Sanger sequencing techniques.

	Patient												Restriction
	P1		P2		P3		P4		P5		P6		enzyme
Genus, species	S	E	S	E	S	E	S	E	S	E	S	E	
*Brevibacterium spp*.									*1*	*6*			*40U*,*SmaI*
*Microbacterium spp*.			*1*	*12*									*10U*, *SpeI*
*Pseudomonas aeruginosa*	*2*	*6*			*3*	*4*	*2*	*2*	*2*	*2*			*20U*, *SpeI*
*Sphingomonas spp*.	*2*	*12*											*10U*,*XbaI*
*Staphylococcus aureus*									*5*	*2*			*40U*, *SmaI*
*Staphylococcus epidermidis*			*1*	*6*							*1*	*3*	*40U*, *SmaI*
*Staphylococcus spp*.			*1*	*7*									*40U*, *SmaI*
*Stenotrophomonas maltophilia*	*1*	*10*					*2*	*5*					*20U*, *SpeI*
*Streptococcus mitis*					*8*	*1*					*2*	*2*	*90U*, *SmaI*
*Streptococcus parasanguinis*					*1*	*1*					*5*	*1*	*90U*, *SmaI*
***Total isolates screened***	*5*	*28*	*3*	*25*	*12*	*6*	*4*	*7*	*8*	*10*	*8*	*6*	

*A total of 122 isolates were screened to determine if isolates are clonally related using pulse field gel electrophoresis (PFGE)*. Organisms that were concordantly identified in both sputum and home environmental isolates and the corresponding number of isolates that were screened. Blank cells represents organisms that were not concordant in a particular patient. P1-P6 refers to patient identification in the study. PFGE was done on each of the concordant isolates using the restrictions enzymes listed.

A large number of organisms from 60 different genera across the six patients were identified (S1 Table). A variety of Gram-positive and Gram-negative organisms with varying oxygen requirements were found to exist in both the home environment and lower airways microbiome (Fig 1 and S1 Table). Not surprisingly, previously unidentified organisms were commonly found in environmental reservoirs (Fig 1 and S1 Table). The most prevalent organism cultured from the home environmental samples by percentage belonged to the *Sphingomonas* genus 15% for patient 1, Family *Micrococcaceae* 19% for patient 2, *Agrococcus* and *Streptococcus* genera—17% of each for patient 3, *Stenotrophomona*s genus 16% for patient 4, Family *Micrococcaceae* 13% for patient 5, Family *Micrococcaceae* 12% for patient 6 (Fig 1 and S1 Table).

Various organisms commonly regarded as normal oropharyngeal flora were cultured from sputum (Fig 1). The most prevalent organisms cultured from sputum samples of patients were common CF microbiota belonging to the *Stenotrophomonas* genus 24% for patient 1, *Streptococcus* genus 35% & 67% for patients 2 and 3 respectively, *Pseudomonas* genus 50% for patient 4, *Staphylococcus* genus 47% for patient 5, and *Streptococcus* genus 41% for patient 6 (Fig 1). The majority of organisms identified from patients 4 and 5 sputum were consistent with chronic *P*. *aeruginosa* and *S*. *aureus* infection, respectively.

**Fig 2:**
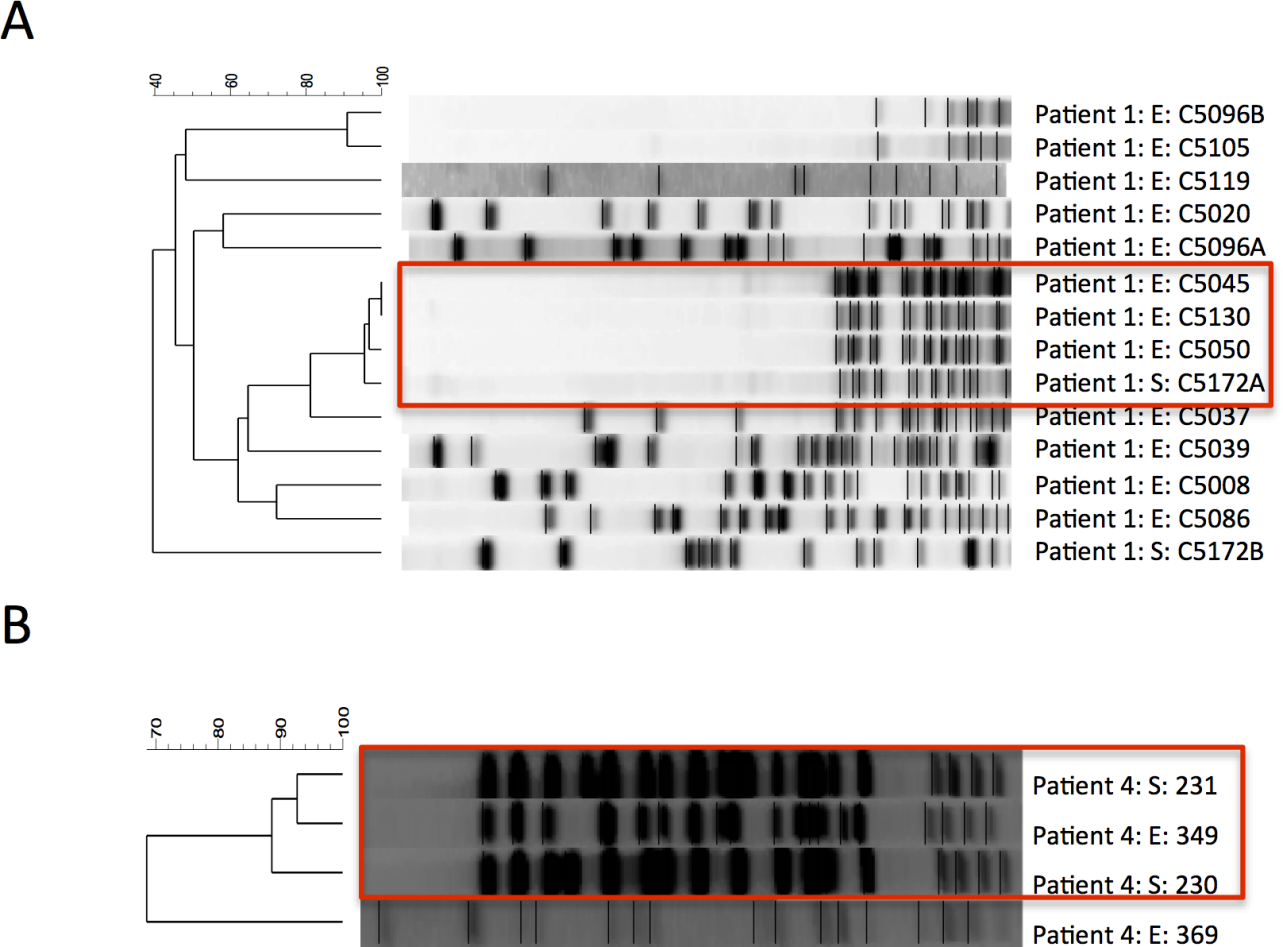
Genotypically related isolates found in the home environment of patients studied. (A) The home environment of patient one (P1) contains genotypically related strains of Sphingomonas species as those found in lower airways. (**B)** The home environment of patient four (P4) also contained an isolate of P. aeruginosa similar to two morphologically distinct isolates collected from their sputum. Genotypically related isolates are highlighted in red. Samples are named as follows Patient ID:S = Sputum isolate /E = environmental isolates:Strain ID number.

A total of 122 isolates from ten species across the six patients were found to be concordant in both home environment and sputum (Table 3). PFGE was carried out using organism specific protocols on all of the concordant isolates collected from sputum and the home environment to determine if environmental strains were genetically related to isolates collected from the lower airways. We identified two situations in which the PFGE patterns demonstrated strain relatedness between environment and lower airways (Fig 2A and 2B). It was impossible to determine based on the retrospective study design if these isolates were the source of lower airways infection, or a consequence of patient related environmental contamination. For Patient 1, three *Sphingomonas* isolates coming from two different environmental sites (sink faucet & shower head) were genetically related to an isolate collected from the lower airways of this patient (Fig 2A). Interestingly, these data also show us that other *Sphingomonas* isolates collected from the various sites of this patient’s home are quite heterogeneous suggesting that there is a wide variety of strains persisting in the home environment. Similarly in Patient 4, the *P*. *aeruginosa* isolates collected from this patient’s sputum was genetically related to the isolate found in the basement sink faucet which they reported to be their principle bathroom (Fig 2B). Despite several concordant species being found between home environments and lower airways, the majority of PFGE patterns did not demonstrate strain relatedness between environment and lower airways (data not shown).

**Fig 3:**
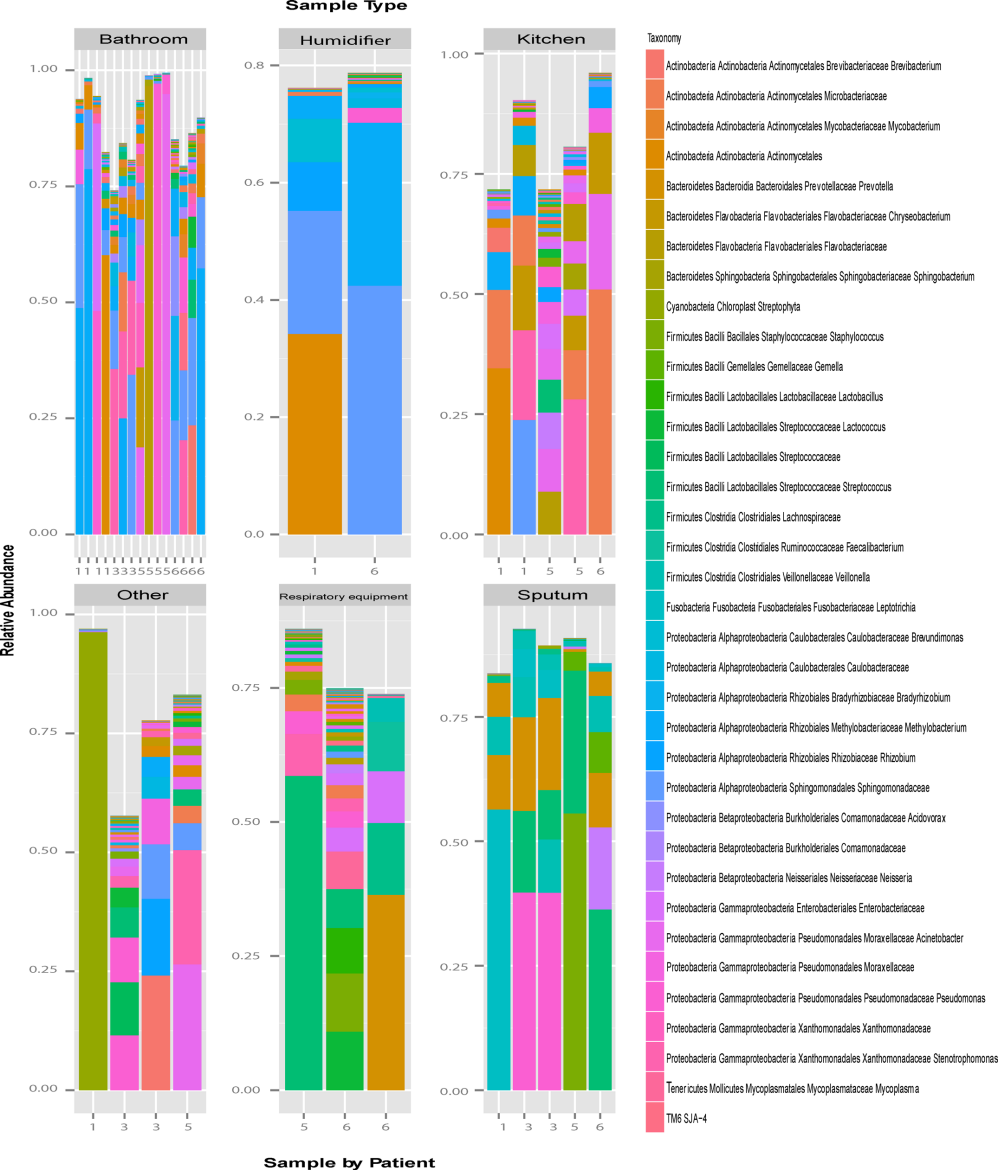
Relative abundance of OTUs present in greater than 1% of samples for 34 of 39 samples collected from patients 1, 3, 5 and 6. Numeric identification on x-axis refers to patient number. Bar plots have been grouped according to sample type. Samples taken from bathroom include bathroom sinks, faucets, drains, and shower heads/hoses. Samples taken from kitchen include sinks, faucets, and drains. Respiratory equipment includes samples taken from patient’s nebulizers and airways clearance device (FLUTTER®, Aptalis). Other samples include samples taken from other respiratory equipment, outside faucets, high efficiency washer, and an aerator.

To assess for the uncultured microbial community of the home, a total of 39 samples taken from the home environment and sputum (from patients 1,3, 5, and 6) were sent for Illumina MiSeq sequencing of the V3 hypervariable region of the 16S rRNA gene. Samples from patients 2 and 4 were not available for assessment in the culture-independent studies. A total of 3,965,211 reads (an average of 116,623.8 reads/sample) for all the samples included in the analysis. Five of the 39 samples were excluded from assessment as they had <1500 reads/sample. Results show that there were 289 uniquely identified genera across all samples from all patients (Fig 3). Samples taken from the home environment and sputum collected from patients 1, 3, 5, and 6 suggest that bathrooms are contaminated by a greater percentage of *Pseudomonas* species compared to other environmental sites. Interestingly, many organisms were not identified by culture and were found using the culture-independent technique, however, in some instances the opposite was also true (Table 4). We speculate that those organisms that were missed in the culture-independent assessments are low abundance organisms that require culture enrichment in order to be detected. This is in accordance with previous findings suggesting that both culture-dependent and culture-independent techniques are required to enhance sensitivity of identifying organisms present in a given sample [6]. For example, using culture-dependent techniques only 9 organisms were cultured from Nebulizers for patients 5 and only 2 organisms were cultured from patient 6. However, using culture independent techniques 7–13 taxa with greater than 1000 reads each were observed (Fig 3 and Table 4). This suggests that perhaps some of the organisms are not stable in this type of environment and the reads represent residual bacterial DNA rather than viable organisms. Indeed, we used a variety of culture conditions to ensure to maximal recovery of potential viable organisms.

**Table 4:** Culture-dependent versus culture-independent microbial contamination of respiratory equipment.

Patient	Patient 5—Nebulizer		Patient 6—Nebulizer	
Taxonomic Identification Family/Genus	Culture dependent profiling	Culture independent profiling	Culture dependent profiling	Culture independent profiling
*Pseudomonas*		**✓**		**✓**
*Lachnospiraceae*		**✓**		**✓**
*Stenotrophomonas*		**✓**		**✓**
*Microbacteriaceae*	**✓**	**✓**		**✓**
*Streptococcus*		**✓**		**✓**
*Staphylococcus*	**✓**	**✓**	**✓**	**✓**
*Lactobacillus*				**✓**
*Lactococcus*				**✓**
*Mycoplasma*				**✓**
*Enterobacteriaceae*				**✓**
*Bifidobacterium*				**✓**
*Sphingobacterium*		**✓**		
*Anoxybacillus*				**✓**
*Bacillaceae*	**✓**		**✓**	**✓**
*Propionicicella*	**✓**			
*Dermabacter*	**✓**			

For culture-independent studies only taxa with greater than 1000 reads were included in this assessment. Taxa with lower quality reads are not included in this table.

*M*. *abscessus* subsp. *abscessus* was one of the organisms that prompted investigation in patients 1 and 3, interestingly we were not able to culture this organism from either patients repeat sputum sample or environmental samples. We were however, able to identify a few *M*. *gordonae* isolates in showerheads of both patients. Similarly in our culture-independent assessments we were able to detect organisms from the *Mycobacterium* genus in both patients homes, however, in low abundance.

Similarly, the newly isolated organisms that prompted investigation in Patient 5 were *P*. *fluorescens* and *S*. *maltophilia* (Table 1) interestingly neither of these organisms were found using culture-dependent techniques in the repeat sputum sample at the time of investigation. However there were four morphologically distinct S. *maltophilia* isolates cultured from samples taken from this patient’s home. Likewise, *A*. *xylosoxidans* was the new organism that prompted investigation in Patient 6, however, this organism was not cultured from either the home environment or the patients repeat sputum assessment. The data herein suggest transient carriage of these organisms could have occurred. Indeed, such transient carriage is reported commonly [48–50]. However, the presence of *S*. *maltophilia* isolates in Patient five’s home suggests that this may be a source for re-infection.

## Discussion

Our understanding of the microbial communities that infect the lower airways in CF remains limited. Whereas clinical microbiology labs have traditionally focused solely on the identification and characterization of a several classical CF pathogens, the adaption of 16s rDNA community sequencing technologies and more extensive culturing approaches have suggested a much greater diversity of organisms infect CF airways [7,10,12,51].

The originating source of lower airways infecting organisms remains poorly understood. Infections can be acquired from a number of possible routes which may be nosocomial; infections acquired from within a healthcare environment through either patient-to-patient or healthcare provider/health care environment-to-patient, or community acquired; infection transmitted from person-to-person outside of the healthcare setting or from an environmental intermediary [15,52]. Mechanisms by which transmission have occurred may be through droplet, aerosol or contact either directly or through fomites [53].

Given its role as the archetypal CF pathogen, environmental sources of acquisition of *P*. *aeruginosa* have been extensively studied.[15,54,55]. Schelstraete *et al*., demonstrated that 72% of bathrooms in the homes of CF patients who were recently identified to have new *P*. *aeruginosa* lower airways infection were also culture positive [15]. However, only 9 of 50 newly infected patients (18%) were identified to have the same colonizing strain of *P*. *aeruginosa* within the bathroom through genotypic analysis [15]. Similar to previous findings our results have shown that sink faucets and drains were sites commonly contaminated by *P*. *aeruginosa* [15,54,55]. We were able to demonstrate genotypic concordance with lower airways of a single isolate of *P*. *aeruginosa* in a newly infected patient suggesting that environmental acquisition of *P*. *aeruginosa* is a rare event. As such, it is likely that specific conditions with respect to the pathogen and host must be met in order to culminate in infection. In addition, the physical conditions of the home environmental sites screened are quite different than the lower airways of CF patients; therefore it is possible that the patients lower airways acts as a selective growth environment for strains that may not be the most abundant in other environmental niches.

When assessed using culture-independent technologies, *Pseudomonas* spp. could be identified in the home of every patient provided enough samples were taken. Latent reservoirs of *P*. *aeruginosa* may be important in CF. In particular, it may be that the approximately 20% of patients that fail to respond to early *P*. *aeruginosa* eradication may be due to re-infection through exposure to a persistent environmental niche [56–60]. If this were true, it may be that clinical outcomes of eradication therapy for *P*. *aeruginosa* may be improved with targeted environmental treatment to reduce *P*. *aeruginosa* burden simultaneous to medical treatment of patients. Regardless of whether the infection originated from the patient or the environment, a persistent untreated environmental source may serve as a residual reservoir enabling re-infection following successful medical therapy of the patient. Furthermore, data from our study and the work of others suggests that the home environment of CF patients does indeed harbor potentially pathogenic organisms beyond just *P*. *aeruginosa*. Organisms such as *S*. *maltophila*, *S*. *aureus*, *Streptococcus* spp., and *Sphingomonas* spp. were also commonly found in patient homes [15,55].

In order to reduce risk of reinfection, strategies of environmental cleaning to remove latent reservoirs of infection may be considered as viable strategies. Such treatments could prospectively be incorporated into early eradication strategies. If targeted treatments were to be routinely advocated, current evidence would suggest that cleaning/replacement of showerheads, and sink faucets would be the highest yield. It is possible in this pilot study that we weren’t able to identify all cases of clonally related isolates in the home environment and lower airway of CF patients due to limitations of sequencing and sampling depth. Perhaps if thousands of isolates were screened from each patients home using even more enhanced culturing conditions clonally linked isolates may have been found at a higher prevalence, although practically this is not feasible.

Less is known about potential sources of other established CF pathogens such as *Stenotrophomonas maltophilia* or *Achromobacter* spp. Results from our culture-independent assessment suggest that there are organisms from the *Stenotrophomonas* genus in high abundance contaminating respiratory equipment of CF patients that were missed in the culture-dependent studies despite a multitude of conditions being attempted. These results are similar to previous findings comparing Sanger sequencing results to deep sequencing results in polymicrobial infections showing that deep sequencing was more discriminating. However, the disadvantage of culture-independent assessments is that bacterial viability cannot be verified. Although we are able to sequence and identify many more organisms using this technique, it is possible that this is actually just bacterial DNA present in the environment rather than viable organisms.

With increasing evidence to support the polymicrobial nature of CF lung disease we took a more broad perspective looking at whether other members of CF lung microbiome may also exist in the home environment. Our results show that other members of the CF lung microbiome including species from the following genera, *Brevibacterium*, *Enterobacter*, *Mycobacterium*, *Neisseria*, *Sphingomonas*, *Staphylococcus* and *Streptococcus* amongst others, are also ubiquitously found in moist sites of the home environment. These organisms may play an important role in CF pathogenesis. For instance Sibley et al. found that the emergence of the *Streptococcus anginosus* group (*S*. *milleri* group) as the numerically dominant organism in sputum was associated with bronchopulmonary exacerbations [9]. While members of CF microbiome were identified in the home environment based on our PFGE results we were only able to identify two instances in which environmental strains were identical to strains collected from the patients sputum samples, suggesting that the majority of these strains are genetically distinct. This again provides support that organisms infecting the airways in CF have specific airway adaptations enabling colonization and persistence, and that not all strains of putative CF pathogens may be capable of causing airways disease in CF.

Precedence exists to suggest that living environment may impact lower airways microbiota in CF. In a recent study done by Hampton et al., they observed that CF siblings residing together had a lower airway microbiota that was much more similar than those living apart [61]. Whereas shared environment is just one factor associated with cohabitating (i.e. intensity of patient-patient exposure, similar diet, similar natural environmental exposure etc.) their findings combined with our own might suggest that home environment may serve as a patient-patient intermediary for microbiota. However, to the best of our knowledge, environmental sources of acquiring bacterial infection in CF have been explored only in regards to specific pathogens and never from a microbiome perspective prior to this work.

Our study has a number of notable limitations, most notably the fact that only six patients were included. However, even in the much larger patient population of 50 patients Schelstraete et al. which focused only on *P*. *aeruginosa*, rarely was a home environmental isolate genotypically related to the respiratory isolate identified [15] Given the frequent identification of CF lung microbiome constituents in the home environment (and the tremendous efforts involved in doing so) and the fact that we were only able to identify genetic similarity in two of the 13 concordant genera assessed it would seem larger scale studies would be impractical. Since our methods of identifying the isolates relied on culture-based techniques and morphological identifiable differences between isolates a sampling bias could have occurred. Furthermore, a broader range of media types could have been used, however, it is unlikely inclusion of more culture conditions beyond the 12 attempted would further increase the recovery of organisms. Our comparison of the microbiota of the home environment and lower airways was performed on only one occasion in each patient–something that may have missed organisms existing transiently in either environment. Using complementary culture-independent studies we did note a significant increase in burden of potential organisms within the environment. However, these culture-independent modalities lack specificity as they are capable of amplification of either free, or dead bacterial DNA, in addition to viable organisms and they thereby potentially overestimate the degree of diversity. Furthermore an exclusive culture-independent approach does not allow for determination of strain relatedness.

Finally, we must point out—we were not able to determine the order of infection; did the patient acquire infection from the home, or did the patient contaminate their home environment? Such criticism exists in all similar studies. However, regardless of the order of infection, an environmental niche of infection was occasionally identified which may serve to re-infect the patient after a successful eradication regimen.

## Conclusions

Where the constituents of the lung microbiota of newly infected CF patients originate remains relatively unknown. We identified that the home environment of CF patients harbors classical CF pathogens such as *P*. *aeruginosa*, *S*. *aureus*, *S*. *maltophilia*, and *Achromobacter* spp, as well as those organisms only recently considered to potentially have a role including members of the genera *Bacteroides*, *Gemella*, *Prevotella*, *Sphingobacterium*, *Streptococcus*, *and Veillonella*. However, the majority of those environmental organisms represent different strains based on PFGE patterns, suggesting that it is rare that the home environment serve as a source of infection within the confines of those reservoirs assessed.

## Supporting Information

S1 Table Proportion of cultured bacteria recovered from home environmental sites and bacterial organism found in patients sputum samples for all 6 patients. (XLSX)
